# Left ventricular apical pacing-induced heart failure in a child after congenital heart surgery: a case report

**DOI:** 10.1093/ehjcr/ytae339

**Published:** 2024-07-10

**Authors:** Rik De Wolf, Roel L F van der Palen, Arend D J ten Harkel, Mark G Hazekamp, Nico A Blom

**Affiliations:** Department of Pediatrics, Division of Pediatric Cardiology, Leiden University Medical Center, Albinusdreef 2, 2333 ZA Leiden, The Netherlands; Department of Pediatrics, Division of Pediatric Cardiology, Leiden University Medical Center, Albinusdreef 2, 2333 ZA Leiden, The Netherlands; Department of Pediatrics, Division of Pediatric Cardiology, Leiden University Medical Center, Albinusdreef 2, 2333 ZA Leiden, The Netherlands; Department of Cardiothoracic Surgery, Leiden University Medical Center, Leiden, The Netherlands; Department of Pediatrics, Division of Pediatric Cardiology, Leiden University Medical Center, Albinusdreef 2, 2333 ZA Leiden, The Netherlands; Department of Pediatrics, Division of Pediatric Cardiology, Amsterdam UMC, Amsterdam, The Netherlands

**Keywords:** Pacing-induced cardiomyopathy, Left ventricular apical pacing, Cardiac resynchronization therapy, Congenital heart disease, Paediatric, Case report

## Abstract

**Background:**

Left ventricular apical pacing (LVAP) is considered to preserve left ventricular (LV) systolic function in both patients with and without congenital heart disease. However, sporadic LVAP-associated cardiac dysfunction in children with complex structural heart disease was recently reported. We present the case of a 2.5-year-old child with complex congenital heart disease and LVAP-induced cardiomyopathy.

**Case summary:**

Corrective surgery for double outlet right ventricle, subpulmonary ventricular septal defect, and transposition of the great arteries was done at the age of 1.5 months. Late complete atrioventricular block occurred, necessitating VVI pacemaker insertion with LV apical epicardial leads. He presented with heart failure and dilated cardiomyopathy 1.5 years after pacemaker insertion and required persistent circulatory support with intravenous inotropes. Speckle tracking echocardiography identified an important LV apical to basal dyssynchrony. After excluding any coronary artery involvement, cardiac resynchronization therapy was performed. Speckle tracking echocardiography guided lead placement resulted in improved LV contraction synchrony. Cardiac function recovered progressively in combination with oral heart failure medication and is almost normal at 10-month follow-up.

**Discussion:**

Right ventricular pacing is a well-known cause of pacing-induced cardiomyopathy. The LV apex and LV free wall are thought to be most optimal locations for ventricular pacing in children. However, LVAP can also be the cause of a pacing-induced cardiomyopathy and decreased systolic LV function in children with complex congenital heart disease due to lack of LV contraction synchrony. Cardiac resynchronization therapy can reverse this LV dysfunction and remodelling.

**Learning points**

- Single-site LV apical pacing (LVAP)-induced LV apical to basal dyssynchrony can lead to LV systolic dysfunction in children with complex congenital heart disease.
- These children warrant a close follow-up of LV systolic function.
- Multisite ventricular pacing with lead placement guided by speckle tracking analysis can improve LV dyssynchrony and reverse LV dysfunction in LVAP.

## Introduction

Left ventricular apical pacing (LVAP) is preferred over right ventricular (RV) pacing in children with complete atrioventricular block requiring an epicardial pacemaker system.^1,2^ Left ventricular apical pacing has shown to preserve global LV function in both children with and without congenital heart disease.^3^ In this case report, we describe LVAP-induced LV dysfunction and heart failure in a child with complex congenital heart disease within 1.5 years after pacemaker implantation. LV function normalized after resynchronization of the LV by placement of an extra basal LV lead, guided by regional wall movement analysis.

## Summary figure

**Table:** 

1.5 months old	Congenital heart surgery for double outlet right ventricle, VSD, and transposition of the great arteries.
11 months old	Late post-operative complete AV block necessitating left ventricular apical pacing.
2 years and 6 months old	Symptomatic heart failure and dilated LV with decreased systolic function requiring hospital admission and start of oral heart failure medication.
2 years and 7 months old	Persistent heart failure despite intensifying treatment to i.v. inotropes for which CRT and pacemaker upgrade to DDD. Subsequently decrease of heart failure medication and hospital discharge.
3 years and 5 months old	Normal LV dimensions, almost normalization of LV systolic function and disappearance of heart failure symptoms.

## Case presentation

**Figure 1:**
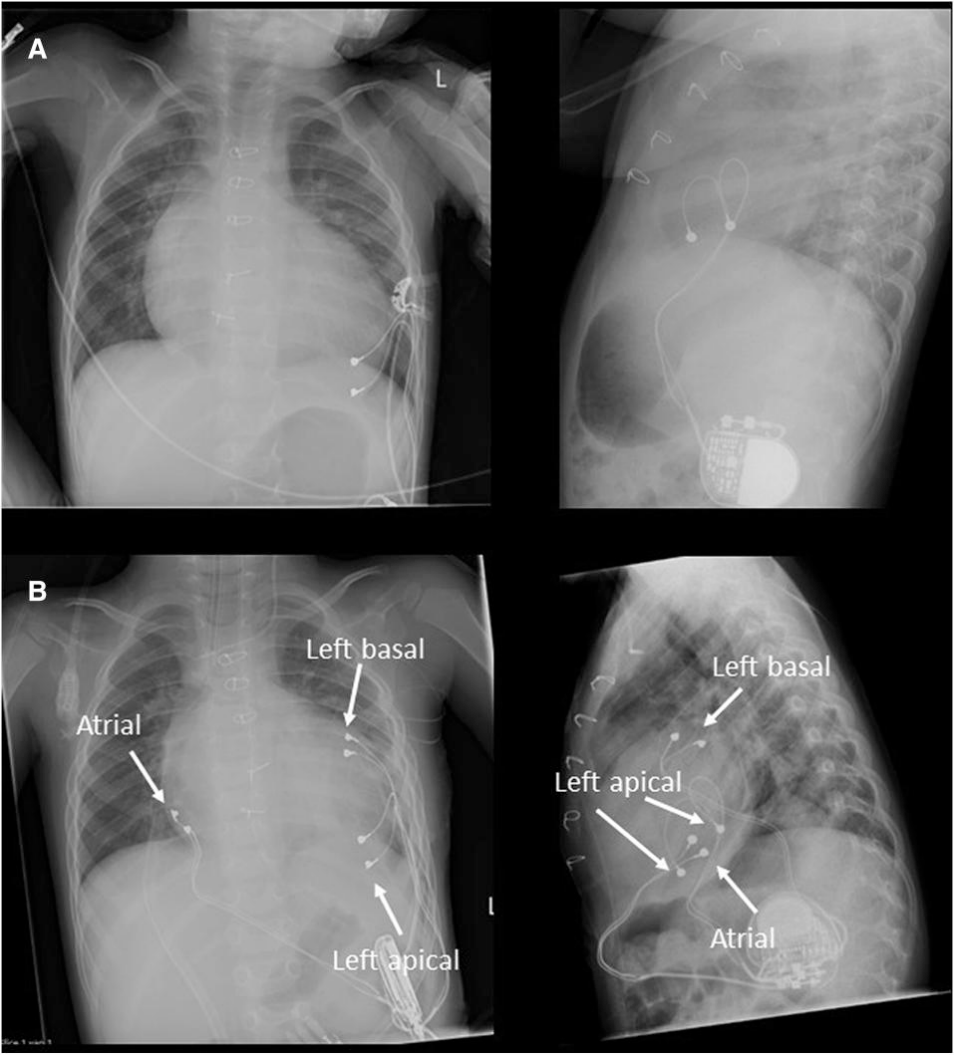
(*A*) Anteroposterior and lateral chest radiograph with apical left ventricular (LV) pacemaker leads after initial pacemaker insertion. (*B*) Anteroposterior and lateral chest radiograph with the original LV leads, additional LV basal free wall, and atrial leads.

A 2.5-year-old boy with complex congenital heart disease had post-operative follow-up at our outpatient clinic. He was born with double outlet right ventricle, large subpulmonary ventricular septal defect (VSD), and transposition of the great arteries with an inverted coronary artery pattern (1R-2LCx, according to the Leiden Convention).^4^ He underwent a successful arterial switch operation and VSD closure at the age of 1.5 months (see Supplementary material online, *Video S1*). A temporary third-degree atrioventricular (AV) block was present post-operatively, which necessitated temporary ventricular pacing for 24 h, with spontaneous recovery to sinus rhythm. A late, symptomatic, third-degree AV block was diagnosed 9 months after surgery for which he received an epicardial pacemaker (MicroPort®—Teo SR) in suprarenal position.^5^ The bipolar steroid eluting electrodes (Medtronic®—CapSure Epi 4968–25 cm) were placed on the LV apex (*Figure 1A*) and the pacemaker was programmed VVIR (lower rate 80 b.p.m., upper rate 185 b.p.m.). He recovered swiftly with good LV function on echocardiography and had regular outpatient follow-up without the need for any cardiovascular drugs.

**Figure 2:**
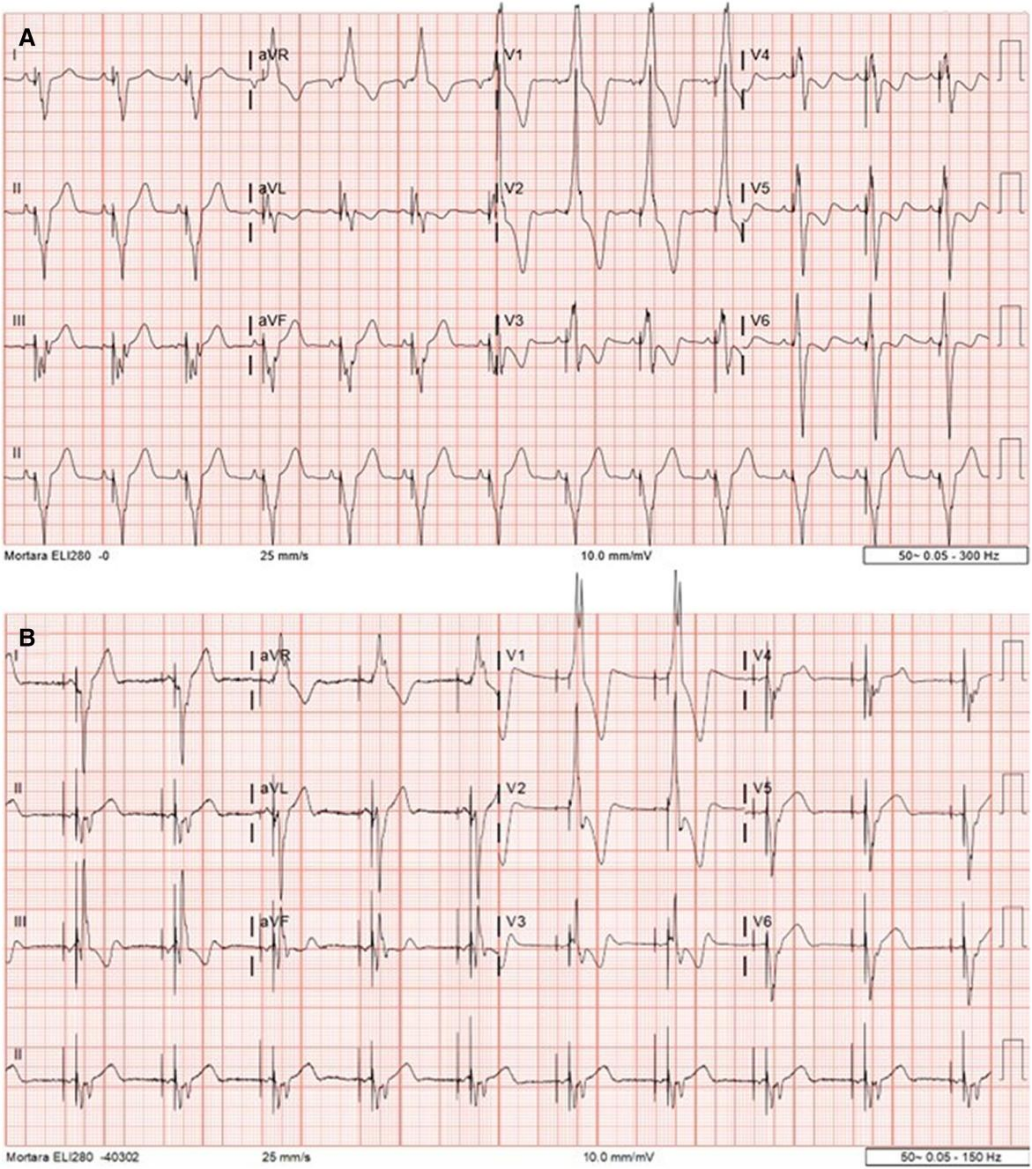
(*A*) Initial ECG with left ventricular apical pacing. The QRS complex has a right superior axis, right bundle branch morphology (RBBB) with QRS duration of 169 ms. (*B*) ECG after cardiac resynchronization therapy. The QRS complex has a slightly more right inferior axis with RBBB morphology and almost unaltered QRS duration of 171 ms.

**Figure 3:**
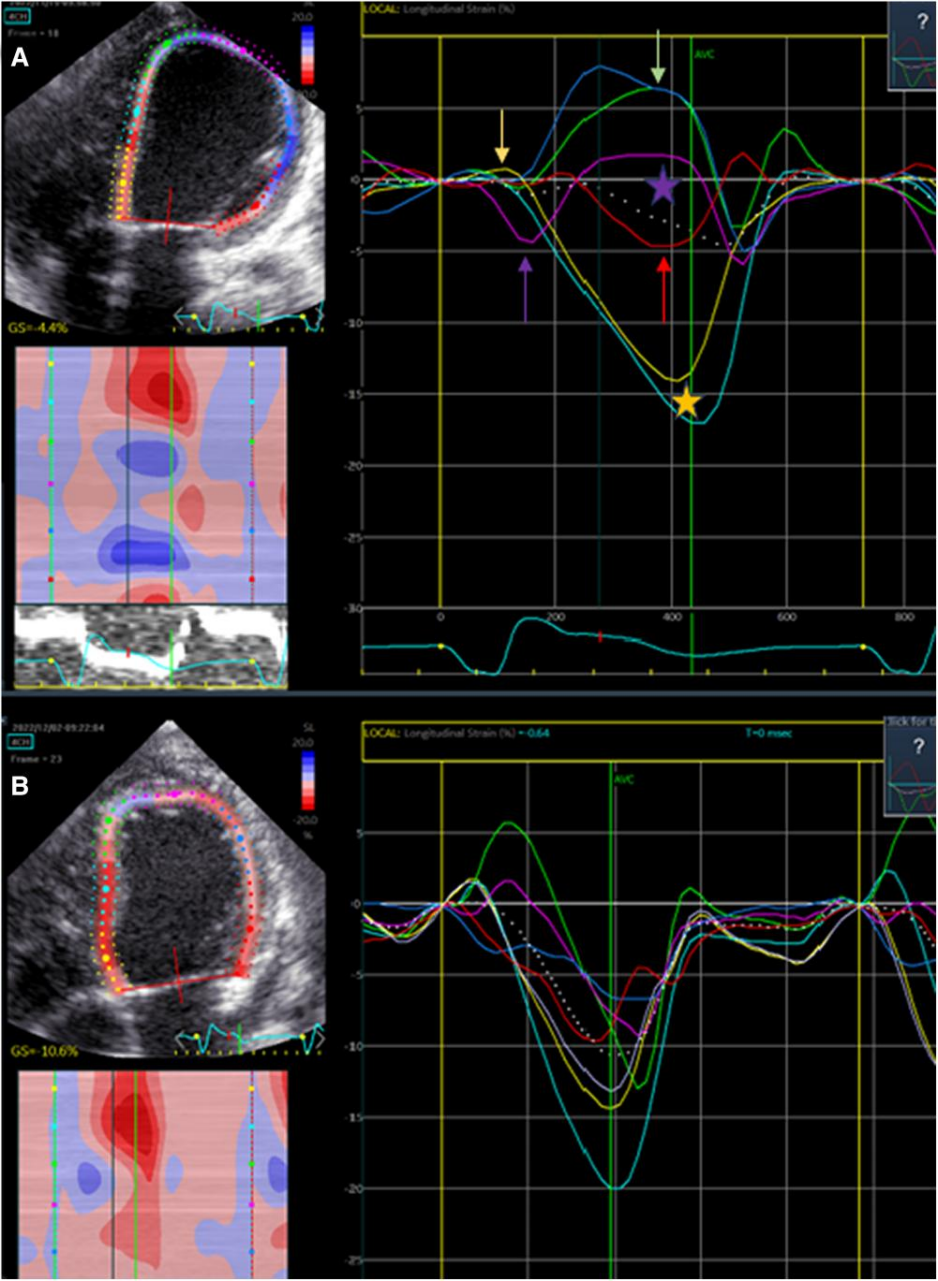
(*A*) Left ventricular (LV) systolic strain curve in the apical four-chamber view before CRT. The apex shows an early contraction (purple arrow) and minimal basal stretch (yellow arrow) with a late rebound stretch (green arrow and purple star) and a basal late contraction (red arrow and yellow star). (*B*) LV systolic strain curve in the apical four-chamber view after CRT with extra lead placement on the LV basal free wall. The LV contracts clearly in a more homogeneous manner with an increased apical to basal synchrony.

The patient presented with fatigue and decreased diuresis. Physical examination at presentation showed a lethargic, pale child, with peripheral oedema and a hepatomegaly. Auscultation showed an II/VI systolic ejection murmur 2nd right intercostal space and no rales. Heart rate was regular 80/min, blood pressure 100/65 mmHg, and respiratory rate 35/min. His growth chart demonstrated no downward deflection. Laboratory testing revealed an increased N-terminal prohormone of brain natriuretic peptide (NT-proBNP) of 22 283 ng/L (upper limit of normal 160 ng/L) and normal troponin T level. The electrocardiogram (ECG) was unaltered with a QRS duration of 169 ms, right superior axis, and right bundle branch block (RBBB) morphology without signs of ischaemia (*Figure 2A*). Echocardiography showed a dilated LV with LV end-diastolic diameter (LVEDD) of 43.2 mm (*z*-score + 2.6) and decreased systolic LV function [ejection fraction (EF) 37%; biplane Simpson method], LV global longitudinal strain (GLS) of −6.4%, moderate tricuspid, and mitral regurgitation. Pacemaker and lead analysis showed normal function (100% LV pacing). A computerized tomography angiogram and coronary angiography were performed to exclude coronary artery pathology and demonstrated patency of the reimplanted coronary arteries. Heart failure therapy was initiated with β-blocker, diuretics, and angiotensin-converting enzyme (ACE) inhibitor. After initial improvement, our patient was readmitted to the paediatric ICU for intravenous (i.v.) milrinone because of increased signs of poor circulation with cold extremities, dyspnoea, and oliguria. He remained dependent of i.v. inotropes, despite additional afterload reduction by ACE-inhibitor treatment and i.v. diuretics. LV function analysis by speckle tracking echocardiography (STE) demonstrated important electromechanical LV apical to basal dyssynchrony (see Supplementary material online, *Video S2*). The LV apical myocardium showed limited early contraction and stretched during late contraction of the LV base at aortic valve closure (‘rebound stretch’) (*Figure 3A*). As LV dyssynchrony was the most plausible cause for the progressive LV failure, he underwent cardiac resynchronization therapy. The optimal lead position was guided by the STE analysis and an extra epicardial lead was placed on the LV basal free wall. An upgrade to a DDD pacemaker system was done (Medtronic® Solara—DDD 60–180 b.p.m.). Different intraventricular delay settings were tested under LV synchrony surveillance by STE. Ultimately, an intra-LV delay of 0 ms showed the best result. LV synchrony on STE improved in comparison to single-site LVAP (*Figure 3B*) and mitral regurgitation decreased. Electrocardiogram showed a RBBB QRS morphology with a slightly more right inferior axis and QRS duration of 171 ms (*Figure 2B*). The patient was weaned off i.v. inotropes one week after CRT. Heart failure medication was continued, NT-proBNP level decreased to 5209 ng/L, and he was discharged 3 weeks later with a LV EF of 42%, a LV GLS of −8.9%, and LVEDD of 42.2 mm (*z* + 2.6). Currently, 10 months after LV resynchronization and heart failure therapy, he is doing well without signs of heart failure with an EF of 54%, LV GLS of −13.9%, normalized LVEDD, and a further decreased NT-proBNP level to 758 ng/L (see Supplementary material online, *Video S3*). The heart failure treatment will be progressively weaned during follow-up.

## Discussion

Atrioventricular block requiring pacemaker therapy occurs in ∼1% of children after congenital heart surgery.^6^ A pacing strategy to preserve cardiac function in this group of patients is important as these patients have a lifelong pacemaker dependency in prospect. In the last decade, growing evidence supports the idea that LV apical or LV lateral wall pacing is superior to RV pacing for chronical single-site pacing in children with either surgical or congenital AV block.^7–9^ It preserves global LV systolic function and septal to lateral synchrony in both children with normal hearts and with congenital heart disease.^3,10^ The LV base is thought to be an inferior pacing site due to an inverse pattern of electromechanical dyssynchrony.^2^

Our patient presented with dilated cardiomyopathy and heart failure 1.5 years after epicardial single chamber LVAP, most likely associated with apical to basal dyssynchrony, which reversed after LV resynchronization through multisite LV pacing. Left ventricular apical pacing is known to induce an apical to basal dyssynchrony, however normally without affecting LV function, although there are limited longer follow-up data on LVAP in patients with complex congenital heart disease.^3^ A recent study by Janousek *et al*. first reported that pacing-induced heart failure can also develop with LVAP, similar as in our patient. They reported three children with complex structural heart disease and dual-chamber LV apical pacemakers who presented with decreased systolic LV function and heart failure. Speckle tracking echocardiography analysis in these patients demonstrated apical to basal dyssynchrony with an early apical contraction and basal pre-stretch, followed by a late basal contraction and apical rebound stretch. CRT directed by STE analysis was performed and led to an increase in contraction efficiency and synchrony with improvement of LV systolic function in two of the three patients.^11^ In our patient, LVAP showed a comparable STE profile, with minimal early apical contraction and basal pre-stretch most likely due to the severity of myocardial impairment. Following a similar approach, CRT was performed by placement of an additional epicardial lead on the LV basal free wall, thereby improving synchrony of the LV myocardial segments as shown by STE. Multisite LV pacing led to prompt disappearance of heart failure symptoms and reversal of LV dysfunction and remodelling during follow-up. As expected, the QRS duration did not significantly change after the procedure, indicating that the total ventricular activation time of RV and LV (electrical dyssynchrony) was not significantly altered, despite improvement of LV mechanical synchrony by multisite LV pacing.

## Conclusion

Our case report emphasizes that in patients with complex congenital heart disease, LVAP can have a deleterious effect on LV function due to apical to basal mechanical delay and LV contraction insufficiency. Multisite LV pacing should be considered in this group of patients either as first line option or if LV dysfunction develops during follow-up.

## Lead author biography


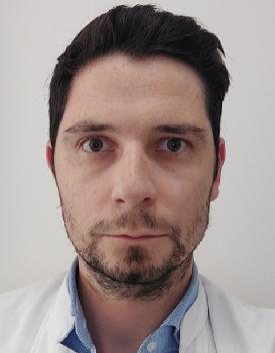


Dr Rik De Wolf is a third-year fellow in paediatric cardiology at the Leiden University Medical Center in The Netherlands. He received his medical degree from the Free University of Brussels and completed his paediatric residency at the University Hospital Brussels in Belgium.

## Supplementary Material

ytae339_Supplementary_Data

## Data Availability

The data underlying this article are available in the article and in its online Supplementary material.

## Notes

**Consent:** Consent for publication has been obtained from the patient’s parents, as he is an underaged child, in line with COPE guidance.

**Funding:** None.
